# Childhood Convulsion: Inquiry about the Concerns and Home Management among Mothers in Tegbesun, a Periurban Community in Ilorin, Nigeria

**DOI:** 10.5402/2012/209609

**Published:** 2012-11-20

**Authors:** Emmanuel Ademola Anigilaje, Omolara Olufunmilayo Anigilaje

**Affiliations:** Department of Paediatrics, Federal Medical Centre, PMB 12245, Benue State, Makurdi, Nigeria

## Abstract

*Background*. The fear and panic felt by most parents when their child convulsed made them apply all sorts of management. *Objective*. This study evaluated the concerns and home management of childhood convulsions among mothers in Tegbesun, a periurban community in Ilorin, Nigeria. *Methods*. A ten-week cross-sectional study comprising 500 mothers interviewed using a structured questionnaire. *Results*. Fear of death was the commonest concern (450, 90%) among mothers. For a witnessed convulsion, the majority took the child to the hospital (414, 82.8%). Cow's urine concoction (74, 87.1%) was the most common item administered to a convulsing child. Putting the hand and/or a spoon into the mouth of the convulsing child was the commonest unwholesome practice (74, 61.2%). None of the subjects safely put the convulsing child on his/her side. *Conclusions*. Maternal concerns are precursors of mismanagement of childhood convulsions, and health education was undertaken at the end of the study.

## 1. Introduction

Convulsion is defined as a paroxysmal involuntary disturbance of brain function that manifests as an impairment or loss of consciousness, abnormal motor activity, behavioral abnormalities, sensory disturbance, or autonomic dysfunction [1]. The abrupt onset of the abnormal motor activities and the accompanying impairment or loss of consciousness is a dreadful and frightening experience to parents who may desperately employ all manners of available therapies in an effort to abort the convulsions [2–6]. While convulsions would have resolved spontaneously with minimal morbidity and mortality as obtained in technologically advanced countries [1], various emergency home therapies employed in developing countries bring about poor outcome [2–6]. The studies by Angyo et al. [2], Fagbule et al. [3], Okoji et al. [7], and Anochie and Graham-Douglas [8] on the knowledge, attitude, and practices (KAPs) of home management of childhood convulsion in Nigeria have been hospital based. The researchers retrospectively surveyed for possible home treatments given to children who were admitted to the hospitals for convulsion. These studies cannot, therefore, be exempted from the pitfalls of such retrospective studies. Ofovwe et al. [9] determined the KAP of home management of febrile convulsions among rural and urban mothers in two Edo villages of south southern Nigeria. However, it is well known that sociocultural beliefs about illnesses are precursors to treatment seeking behavior and as such the findings of Ofovwe et al. [9] would probably reflect mothers' belief about childhood convulsion in the Edo villages. The present study, therefore, determined for the first time, the concerns of mothers about childhood convulsions and also underscored the various home preparations and interventions applied to treat childhood convulsion by mothers in Tegbesun, a periurban community in Ilorin, north central Nigeria. It brought awareness to harmful practices that are being employed to treat convulsions at home in a rural community that is continuously interfaced with the civilization of urban living. 

## 2. Materials and Methods

It was a six-week descriptive cross-sectional study that was carried out between February and March 2010 in Tegbesun, a rural community situated about 4 kilometers from Ilorin, capital of Kwara State in north central Nigeria. Tegbesun is one of the communities designated for Community-Based Experience and Services (COBESs) for the medical students of the University of Ilorin and remained a research community of the Department of Epidemiology and community of the same university. The study population consisted of mothers (18 years and above) who are often at home to care for children who are sick. A minimum sample size of 384, for the study was calculated using the formula

(1) $$\begin{array}{c} N=\frac{{\left(Zi-\alpha \right)}^{2}\left(P\right)\left(1-P\right)}{d^{2}}, \end{array}$$

where *Zi* − *α* = 1.96 (a standard normal deviate), *P* = 0.50 (the proportion in the target population estimated to have a particular characteristic), *d* = 0.05 (proportion of sampling error tolerated).

However, to accommodate for a nonresponse rate of 20%, the sample size was rounded off to 500.

A systematic random sampling was employed. The Primary Health numbers on all the houses in the community were listed. The sampling interval for the houses was calculated by dividing the number of houses by the sample size of 500. Selection of all eligible mothers in the selected houses was done until the sample size was obtained. Revisitations of selected houses were done when subjects were absent at the initial visits.

The research instrument was a structured questionnaire. It was emphasized that anyone was at liberty to decline participation, and participants were also free to withdraw at any stage of the study. The questionnaire was pretested among 50 adult females in Ogidi, an adjacent community in the same local government area for validity and reliability. The pre-tested questionnaire was analyzed and the necessary corrections were made. The survey team consisted of 6 female medical students of the University of Ilorin. These students were fluent in Yoruba language and were also familiar with the culture of the community. They were trained for the administration of the questionnaires adapted in Yoruba language and their proficiency was verified through role-play and pretesting. 

Data analysis was done to produce frequency distribution tables.

## 3. Results

A total of 500 women between the ages of 18 and 65 years were interviewed. The mean age was 35.5 ± 4.4 years, with the majority falling within 18 and 47 years (Table 1). The majority (473) of the subjects were married (94.6%) and a majority (406) were also Muslims (81.2%). The majority (495) of the subjects (99%) were Yorubas, and four subjects (0.8%) were Ibos. Only one Hausa woman was found among the subjects. Other characteristics of the subjects are shown in Table 2. The most common cause of concern among subjects was the fear of death (450, 90%), followed by fear of reoccurrence (389, 77.8%), mental retardation (290, 58%), physical disability (100, 20.0%), and visual impairment (57, 11.4%) (Table 3). For the immediate action taken when a child is convulsing, the majority agreed upon taking the child to the hospital (414, 82.8%), followed by calling for help (408, 81.6%) (Table 4). Among the 85 subjects who have managed convulsions at home, cow's urine concoction (74, 87.1%) was the most common item administered to a convulsing child. Thirty-eight mothers (44.7%) kept these items at home, while the majority (47, 55.3%) did not keep these items at home, rather collected them from neighbors (33, 70.2%), herbalist (13, 27.7%), and a passerby (1, 2.1%). Also among the 85 subjects that have managed convulsion at home, putting the hand and/or the spoon into the mouth of the convulsing child is the most common practice among the subjects (74, 61.2%), followed by making scarifications on the face/body of the convulsing child (20, 16.5%), recitation of the holy Quran (15, 12.4%), burning of the child's extremities in open fire (10, 8.3%), and recitation of incantations (2, 1.7%). None of the subjects correctly and safely put the convulsing child to lie on his/her side, while the convulsion lasted (Table 5). Seventy-one subjects have witnessed side effects including aspiration of liquid items (45, 63.4%), damage to the eyes (23, 32.4%), burns/contracture of the extremities (2, 2.8%), and death (1, 1.4%) (Table 6).

## 4. Discussion

In this study, the most common concern expressed among subjects was the fear of death (450, 90%). This finding was similar to that of Parmar et al. [10] in India who also reported that 90% of the parents worried about possible demise of the convulsing child. However, Kolahi and Tahmooreszadeh [11] in Iran reported a lower proportion (33%) of mothers, and similarly Kurugol et al. [12] in Turkey reported 38.5% of the child's parents nursing the fear of death for the convulsing child. The abrupt onset of the abnormal motor activities and the accompanying impairment or loss of consciousness is a dreadful and frightening experience and may readily explain the parental concern of imminent death. Furthermore, 389 subjects (77.8%) feared a recurrence of convulsion, a finding that was lower than that of Kurugol et al. [12] (91.4%), but higher than that of Parmar et al. [10] that reported a fear of recurrence in 19.3% of their subjects. A majority of the subjects, 414 (82.8%), would immediately take a convulsing child to the hospital whilst a similar majority, 408 (81.6%), would call for help from their neighbors who may then assist them in taking the convulsion child to the hospital or offer remedies to abort the convulsion. Twenty-two subjects (4.4%) would seek assistance of the herbalist. This option of seeking for the herbalist may reflect the beliefs of these subjects in the traditional herbal cure for childhood convulsion. In the socio-cultural milieu, traditional beliefs are the basis for local definitions of health problems occurring in the community as to whether health issues are due to natural causes, spirits, or bewitchment [13]. Thus, treatment seeking for childhood convulsion depends very much on the belief of whether a natural or an unnatural cause is responsible for childhood convulsion [13]. Only eighty-five subjects (17.0%) agreed to have treated convulsion at home. This proportion of subjects that have treated convulsion at home in this study was, however, lower than 28% reported by Anochie and Graham-Douglas [8] in Port Harcourt, 65% by Okoji et al. [7], also in Port Harcourt, 60.2% by Fagbule et al. [3] in Ilorin, 67.5% by Angyo et al. [2] in Jos, and 100% of the rural women in the study of Ofovwe et al. [9] in Edo. Whilst the difference in the proportion of subjects that have treated convulsions at home may reflect the setting of these studies, that is, community based in present study against hospital-based studies of Anochie and Graham-Douglas [8], Okoji et al. [7], Fagbule et al. [3], and Angyo et al. [2]. However, it may not explain why all the rural women in the study of Ofovwe et al. [9] attested to the use of home remedies for childhood convulsions.

Among women who agreed to have treated convulsion at home in the present study, cow's urine concoction was the most notorious item (87.1%). This finding was in agreement with that of Fagbule et al. [3] who also reported the use of cow's urine in seventy-one (60.2%) of the 118 cases questioned. The use of cow's urine concoction to abort childhood convulsion among the Yorubas of south western Nigeria has been reported earlier [3, 6], and cow's urine was either forced into the prised mouth of the convulsing child with the hope that he would swallow it or the concoction is painted topically on the body of the convulsing child. The ingredients used in preparing cow's urine concoction were first published in 1964 by Atalabi [14]. The concoction is made from tobacco leaves, garlic leaves, basil leaves, lemon juice, rock salt, and bulb of onion, all soaked in cow's urine for varying length of time. In other cultures in Nigeria and in Africa, human urine is either offered to convulsing child to drink as reported by Ofovwe et al. [9] in Edo state, southern Nigeria or the mother directly urinates on the child as reported by Makundi et al. [13] in Tanzania. Cow's urine concoction, on its own gives rise to various complications including severe hypoglycemia, generalized spasticity, decerebrate rigidity, choreoathetosis, cortical blindness, and convulsion [6]. It is, therefore, ironical that an item employed to control convulsion also has the potential of causing convulsion. Furthermore, daily administration of cow's urine concoction as a prophylaxis for convulsion to children in the same household or compound is a common practice in a rural Yoruba community. It is a common place to see children queuing up to swallow their portions of cow-urine concoction and showing off their “manliness” by their refusal to facially express the bitter taste of the concoction. Outside Africa, traditional herbal concoctions have also been used to treat convulsion. In Japanese Kampo medicine [15], TJ-960 has been found to have reliable anticonvulsing and cognitive improving effects among epileptic patients. In Indian Ayurvedic medicine [16], Ashwagandha, Brahmirasayan, and Brahmigritham have been used for centuries to control seizures. 

 Other items, including palm oil (65.9%), cereal gruels (56.5%), onions to the eyes (38.8%) and alligator pepper (20.0%), were also used by the eighty-five women who have treated convulsion at home in the present study. In Tanzania, Makundi et al. [13] also reported that mothers used elephant's dung smoke, while in Edo state, southern Nigeria, kerosene and petrol applications, were common [9]. It thus appears as if the items used for aborting childhood convulsion vary from one community to another and within households in the same community.

 A majority of the mothers (44.7%) that have treated childhood convulsion at home claimed not to have kept such items at home. Although one may be tempted to expect a higher percentage of mothers to keep such items at home, that expectation should be judged in the socio-cultural context of many African societies whereby keeping medications at home is regarded as an invitation to “mysterious” illness like convulsion. Neighbors (70.2%), herbalists (27.7%), and a passerby (2.1%) were other sources of such items. 

 None of the subjects acknowledged the correct practice of putting the child safely on his or her side. This finding differed from the study of Deng et al. [17] whereby 9.5% of subjects and that of Rutter and Metcalfe [18] where 16% of subjects acknowledged this practice. It is well known that children with convulsion are expected to be laid safely on their side to prevent injury resulting from falls and dangerous aspiration of secretion that may be regurgitated into their mouths.

 The concept of putting hand and/or spoon in the mouth (61.2%) appeared to be more wide spread among the subjects than other practices including making scarifications (16.5%), recitation of the holy Quran (12.4%), burning of the extremities in fire (8.3%), and incantation (1.7%) to cast away the “evil spirit” and/or to appease “angry gods.” In the Yoruba culture, it is believed that when a child is convulsing, all efforts must be put in place to prevent the child from gnashing the teeth, an event that may lead to instant “demise” of the convulsing child. Thus, any item including the parent's hand, which is most readily available, is put into the mouth of the convulsing child. Other items including spoon, stick, and in some cases small pestle are quickly placed in the mouth of a convulsing child to “prevent” death. Furthermore, the concept of making therapeutic incisions on a convulsing child is also a common practice in Yoruba land. It is believed that childhood convulsions like other illnesses including sickle cell anaemia and arthritis are due to “black blood” and incision making create an avenue for letting out this “black blood” and thus affect healing. Some subjects (8.3%) also burned the feet of the child in an open fire. This was lower than 21.4% as reported by Anochie and Graham-Douglas [8] in Port-Harcourt among 50 children with convulsion but contrasted sharply with the reports of Makundi et al. [13] and Ofovwe et al. [9] who did not find this mode of treating convulsion in their own series. This mode of treatment (burning an extremity in fire) thus appears as one of those traditional practices that have been passed down from one generation to another, without accompanying logical explanation. However, this mode of burning the extremities closely simulates the practice among women in Enugu, south eastern Nigeria whereby women often cover up febrile children with warm clothing [4]. Furthermore, some women attested to the help of an Islamic scholar who recited some verses of the holy Quran in order to abort the convulsion. This reason for the recitation of the holy Quran differed from the reason explained by Makundi et al. [13] in Tanzania whereby the holy Quran was read as a form of divination to identify the exact cause of the convulsion so that appropriate treatment may then be given.

Choking and aspiration pneumonitis (63.4%) were the commonest side effects witnessed at home. Other complications included eye damage (32.4%) contractures (2.8%) from burns injury and one death (1.4%). These findings are not unexpected in the light of the various modes of treatment employed in the home management of childhood convulsions in this study. Similar complications were reported by Anochie and Graham-Douglas [8], Fagbule et al. [3], and Angyo et al. [2]. 

## 5. Conclusion

The present study had revealed beyond reasonable doubts that there was a paucity of correct and adequate knowledge of childhood convulsion. It brought to the fore various harmful practices that mothers applied to treat childhood convulsion at home. At the completion of the study, appropriate health information was given to the parents regarding what they needed to know about childhood convulsions, its causes, prevention and management. Women need to know that convulsion on its own is not out-rightly dangerous. Simple laying of a convulsing child on the side as an emergency measure was emphasized during the health information session. 

## Figures and Tables

**Table 1:** Age group of subjects.

Age group	No.	%
18–27	120	24
28–37	200	40
38–47	96	19.2
48–57	64	12.8
≥58	20	4

Total	500	100

**Table 2:** Some demographic characteristics of subjects.

Characteristics	No.	%
Marital status		
Single mothers	2	0.4
Divorced	7	1.4
Widowed	18	3.6
Married	473	94.6

Total	500	100

Religion		
Christianity	94	18.8
Islam	406	81.2
Traditional religion	—	—

Total	500	100

Education		
None	167	33.4
Primary	168	33.6
Secondary	155	31.0
Tertiary	10	2.0

Total	500	100

Occupation		
Farming	320	50.5
Trading	272	42.9
House wives	25	3.9
Civil servants	17	2.7

Total	500	100

Ethnicity		
Yoruba	495	99.0
Ibo	4	0.8
Hausa	1	0.2

Total	500	100

**Table 3:** Concerns of mothers when a child is convulsing.

Concerns	Yes (%)	No (%)	Do not know (%)	Total (%)
Fear of death	450 (90.0)	15 (3.0)	35 (7.0)	500 (100)
Fear of reoccurrence	389 (77.8)	37 (7.4)	74 (14.8)	500 (100)
Fear of mental retardation	290 (58.0)	150 (30.0)	60 (12.0)	500 (100)
Fear of physical disability	100 (20.0)	350 (70.0)	50 (10.0)	500 (100)
Fear of visual impairment	57 (11.4)	389 (77.8)	54 (10.8)	500 (100)

**Table 4:** Action taken when a child is convulsing.

Action taken	Yes (%)	No (%)	Total (%)
Take the child to the hospital	414 (82.8)	86 (17.2)	500 (100)
Call for help	408 (81.6)	92 (18.4)	500 (100)
Apply home treatments	85 (17.0)	415 (83.0)	500 (100)
Take the child to the herbalist	22 (4.4)	478 (95.6)	500 (100)

**Table 5:** Items used for home treatment of convulsions.

Items used	Yes (%)	No (%)	Total (%)
Items used			
Cow's urine concoction	74 (87.1)	11 (12.9)	85 (100)
Palm oil	56 (65.9)	29 (34.1)	85 (100)
Cereal gruel	48 (56.5)	37 (43.5)	85 (100)
Onion applied to the eyes	33 (38.8)	52 (61.2)	85 (100)
Alligator pepper applied to the eye	17 (20.0)	68 (80.0)	85 (100)
Others	Nil	Nil	Nil
Items kept at home	38 (44.7)	47 (55.3)	85 (100)
Sources of items not kept at home			
Neighbors	33 (70.2)	14 (29.8)	47 (100)
Herbalist	13 (27.7)	34 (72.3)	47 (100)
A Passerby	1 (2.1)	46 (97.9)	47 (100)
Others	Nil	Nil	Nil
Practices			
Put hand/spoon into the child's mouth	74 (61.2)	47 (38.8)	121 (100)
Scarification	20 (16.5)	101 (83.5)	121 (100)
Recitation of the holy books	15 (12.4)	106 (87.6)	121 (100)
Burning of the child's extremities in open fire	10 (8.3)	111 (91.7)	121 (100)
Incantation	2 (1.7)	119 (98.3)	121 (100)
Safely put the child to lie on the side	— (—)	— (—)	— (—)

**Table 6:** Side effects witnessed at home.

Side effects witnessed	Yes	No	Total (%)
Aspiration	45 (63.4)	26 (36.6)	71 (100)
Eyes' damage	23 (32.4)	48 (67.6)	71 (100)
Burns/contractures	2 (2.8)	69 (97.2)	71 (100)

## References

[1]  Haslam RHA, Behrman RE, Kliegman RM, Jenson HB (2000). The nervous system. *Nelson Textbook of Paediatrics*.

[2] Angyo IA, Lawson JO, Okpeh ES (1997). Febrile convulsions in Jos. *Nigerian Journal of Paediatrics*.

[3] Fagbule D, Chike-Obi UD, Akintunde EA (1991). Febrile convulsions in Ilorin. *Nigerian Journal of Paediatrics*.

[4] Izuora GI, Azubuike JC (1977). Prevalence of seizure disorders in Nigerian children around Enugu (a preliminary report). *Central African Journal of Medicine*.

[5] Iloeje SO (1989). The impact of socio-cultural factors on febrile convulsions in Nigeria. *West African Journal of Medicine*.

[6] Osuntokun BO (1969). Convulsive disorders in Nigerians. *East African Medical Journal*.

[7] Okoji GO, Peterside IE, Oruamabo RS (1993). Childhood convulsions: a hospital survey on traditional remedies. *African journal of medicine and medical sciences*.

[8] Anochie I, Graham-Douglas IB (2000). Non-accidental injuries associated with convulsions in Port Harcourt, Nigeria. *Anil Aggrawal’s Internet Journal of Forensic Medicine and Toxicology*.

[9] Ofovwe GE, Ibadin OM, Ofovwe EC, Okolo AA (2002). Home management of febrile convulsion in an African population: a comparison of urban and rural mothers’ knowledge attitude and practice. *Journal of the Neurological Sciences*.

[10] Parmar RC, Sahu DR, Bavdekar SB (2001). Knowledge, attitude and practices of parents of children with febrile con vulsion. *Journal of Postgraduate Medicine*.

[11] Kolahi AA, Tahmooreszadeh S (2009). First febrile convulsions: inquiry about the knowledge, attitudes and concerns of the patients’ mothers. *European Journal of Pediatrics*.

[12] Kurugol NZ, Tutuncuoglu S, Terkgul H (1995). The family attitudes towards febrile convulsions. *The Indian Journal of Pediatrics*.

[13] Makundi EA, Malebo HM, Mhame P, Kitua AY, Warsame M (2006). Role of traditional healers in the management of severe malaria among children below five years of age: the case of Kilosa and Handeni districts, Tanzania. *Malaria Journal*.

[14] Atalabi O (1964). ‘Cow's urine’ poisoning. *Dokita*.

[15] Nagabuko S, Niwa SI, Kumagai N (1993). Effects of TH-960(SK) ON Sternberg's paradigm results in epileptic patient. *The Japanese Society of Psychiatry and Neurology*.

[16] Kulkarni SK, Verma A (1993). Protective effect of BR-16A (Mentat), a herbal preparation on alcohol abstinence-induced anxiety and convulsions. *Indian Journal of Experimental Biology*.

[17] Deng CT, Zulkifli HI, Azizi BH (1996). Parental reactions to febrile seizures in Malaysian children. *Medical Journal of Malaysia*.

[18] Rutter N, Metcalfe DH (1978). Febrile convulsions-what do parents do?. *British Medical Journal*.

